# *In situ* fixation and subsequent collection of cultured endothelial cells in a shear flow

**DOI:** 10.1016/j.mex.2019.05.001

**Published:** 2019-05-03

**Authors:** Jessica L. Aldrich, David S. Long

**Affiliations:** Department of Biomedical Engineering, Wichita State University, Wichita, KS, USA

**Keywords:** *In situ* fixation and subsequent collection of cultured endothelial cells in a shear flow, Endothelial cells, Endothelium, Adherent cells, *In situ* fixation, Cell dissociation, Mechanogenomics, Hi-C, Chromatin conformation capture

## Abstract

*In situ* fixation of adherent cells is a necessary process for downstream assays. Current methods to dissociate adherent endothelial cells require the use of a cell scraper that may introduce variability in nuclear morphology. Also, a cell scraper is not an option for experiments using sealed flow chambers. HMEC-1 cells were sheared at 5 dyn/cm^2^ for 24 h and then fixed *in situ*, quenched, and dissociated at the same shear rate. Analysis revealed no statistically significant change in nuclear shape between the steps of fixation and dissociation. This method outlines an alternative for the dissociation of adherent sheared endothelial cells after being fixed *in situ* in a micro-scale channel without causing a change in the nuclear morphology.

• This method can be used with any commercially available, or custom-made, flow chamber and flow system.
• Allows for downstream experimentation with adherent cells fixed *in situ*, such as Hi-C analysis, without impacting nuclear morphology or chromatin organization.
• Cells are cultured, fixed, and dissociated at the same shear rate. Using the same shear rate for each step yields results that are not influenced by variable forces.

## Graphical abstract

**Figure:**
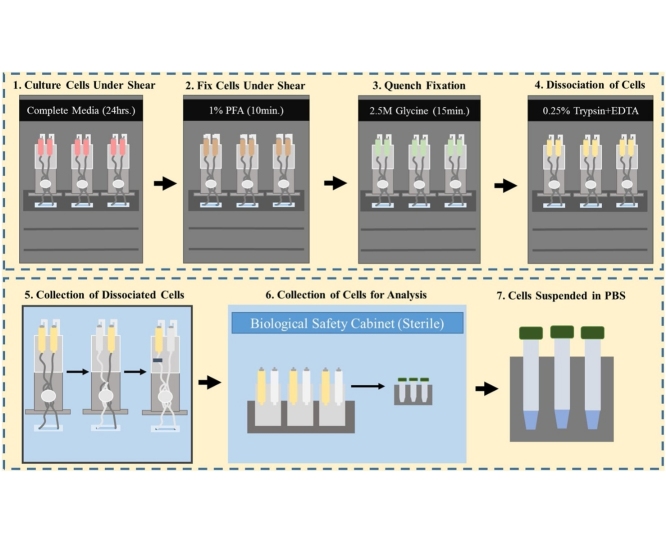

**Specifications Table**

**Table:** 

Subject Area:	*Engineering*
More specific subject area:	*Mechanobiology and Biomedical Engineering*
Method name:	*In Situ* Fixation and Subsequent Collection of Cultured Endothelial Cells in a Shear Flow
Name and reference of original method:	*N/A*
Resource availability:	*All resource information needed to reproduce this method is integrated in the paper (i.e. reagent names, equipment, software).*

## Method details

### Overview

The cellular microenvironment plays a substantial role in cellular function and genome regulation [1]. Mechanogenomics describes the role the mechanical forces have on the expression of the nuclear genome. This area encompasses aspects such as substrate stiffness on nuclear shape [2], flow mediation on cellular structure and function [3], force induction on chromatin morphology [4]. One tool for studying mechanogenomics and the three-dimensional architecture of genomes and chromatin interaction is chromatin conformation capture, specifically Hi-C [5,6].

Hi-C analysis is a high-throughput method of chromatin conformation capture used to analyze the 3-dimensional chromatin within the nucleus [6]. Hi-C develops a genome map by analyzing chromatin interactions and topologically associated domains (TADs). Completing Hi-C analysis relies on cross-linking the DNA *in situ* so the sequences will reflect the biological or experimental conditions under consideration. Cross-linking DNA *in situ* can easily be performed for cells cultured in suspension. In contrast, adherent cells are absorbed to a growth surface by a molecular mechanism that involves the cytoskeleton, which has an effect on nuclear and cellular morphology. The linkage of nuclear morphology and cellular morphology suggests that crosslinking adherent cells while they are still adhered to the culturing surface is necessary to preserve global nuclear organization [7]. Typically, adherent cells cultured in culture plastic-ware (*e.g.*, flasks, dishes, welled plates) are fixed *in situ* and removed from the culture dish using a cell scraper [[8], [9], [10]]. Although scraping can effectively remove adherent cells, this technique introduces variation of the external force on the cell [11]. Moreover, the scraping process affects cells differently based on two parameters: (1) the mechanical stiffness of the cell line; and, (2) the force used to remove the cells from the growth surface, which may vary substantially between experimenters and between experiments. These two parameters can have negative downstream implications in the development of a Hi-C library for adherent cells. Current methods for preparing cells for Hi-C analysis rely on fixing cells in suspension [12]. In fact, the removal of adherent cells is often not addressed in protocols relating to the preparation of these cells for Hi-C analysis [13]. However, the need to fix *in situ* and collect adherent cells that reduces variability has been discussed [2].

Protocols to fix *in situ* and collect adherent cells are further complicated for some adherent cell types. Many types of adherent cells (*e.g.,* endothelial cells, alveolar epithelial cells, kidney cells) are exposed to fluid shear under normal physiological conditions. Thus, to mimic the native environment of these cells, a physiologically-realistic fluid shear is typically applied during culture. To apply this fluid shear, a pump system and a parallel-plate, or similar, flow chamber can be used [3,14]. This experimental setup adds an additional challenge to fixing the cells *in situ*, dissociating, and subsequently collecting. Collecting these cells by scraping is not possible because the enclosed (sealed) flow chambers have dimensions on the millimeter- to micron-scale; thus, scrapers cannot access the growth area. As a result, cell scraping is not a viable option for cells grown in flow chambers. Therefore, an alternate technique is required.

Endothelial cells in the cardiovascular system are continually exposed to fluid shear stress as blood pulses through the body. Changes in shear stress may be a factor in the development of cardiovascular disease [15,16]. Cardiovascular disease is a leading cause of non-accidental death across the United States. This disease manifests in regions of the vasculature with branch points (*e.g.*, carotid bifurcation) and curves (*e.g.,* aortic arch) where oscillatory blood flow occurs (*i.e.,* non-uniform/irregular distribution of low shear stress). This oscillatory flow affects the hemodynamic force experienced by the cells lining the blood vessels (endothelial cells). Previous experimental studies have shown that fluid shear stress can alter endothelial cell morphology [3,17,18] and endothelial gene expression [19,20]. Endothelial cells in disturbed flow are unable to return forces within the cell to equilibrium, which is an underlying early cause of endothelial-cell related disease. Moreover, the inability to return these forces to equilibrium can activate endothelial cells to initiate cardiovascular disease [18,21]. Using Hi-C, the chromatin reorganization occurring in endothelial cells exposed to fluid shear stress can be studied. Currently, to our knowledge, there is no effective way to study changes in nuclear shape or chromatin organization in adherent endothelial cells that are fixed *in situ*. The purpose of this protocol is to develop an effective method to collect adherent endothelial cells to be used for Hi-C analysis.

In this paper we present a method for fixing adherent sheared endothelial cells *in situ* and collecting these cells under the same shear forces used during the experiment for Hi-C analysis. This method allows adherent cells to be collected while maintaining both the morphology of the cells and nuclei developed during exposure to fluid shear stress.

## Method procedure

### Cell culture

Human Microvascular Endothelial cells (HMEC-1, #CRL-3243, P28, ATCC, Manassas, VA) were maintained in complete growth media. Complete growth media consisted of MCDB 131 media (#10372019, Gibco, Grand Island, NY) supplemented with low-serum growth supplement (#S-003-10, concentration: FBS, 2% (v/v); hydrocortisone, 1 μg/ml; human epidermal growth factor, 10 ng/ml; basic fibroblast growth factor, 3 ng/ml; and heparin, 10 μg/ml), penicillin/streptomycin (at 100 U/ml and 100 μg/ml concentration, respectively #15-140-122, Thermo Fisher Scientific), l-Glutamine (200 mM, #25030081, Gibco), and, FBS (#15000044, Thermo Fisher Scientific) was added to result in a 10% (v/v) final concentration. The cells were incubated in a 37 ^○^C incubator with 5% CO_2_.

A culture of HMEC-1 cells was prepared in a T75-ml flask following standard cell culture procedures [22]. Materials needed for the cell perfusion portion of the study were placed in the CO_2_ incubator for 24 h prior to experimentation to equilibrate and de-gas. These materials included the following: (1) an Ibidi Fluidic unit; (2) 1 Ibidi Sticky Slide I^0.4^ Luer Lock slide with #1.5 IbiTreat polymer coverslip (#80178 and #10814, respectively, Ibidi, Martinsried, Germany); (3) 4 red perfusion sets (#10962, Ibidi) (1 sterile set, 3 autoclaved sets); (4) 4 Reservoir sets (#10971, 12 ml, Ibidi); (5) 20 ml complete growth media; and, (6) a small silicone mat for the slide. The #1.5 IbiTreat polymer coverslip was sterilized with 70% Ethanol and attached to the Ibidi Sticky Slide prior to placement in the CO_2_ Incubator.

**Fig. 1:**
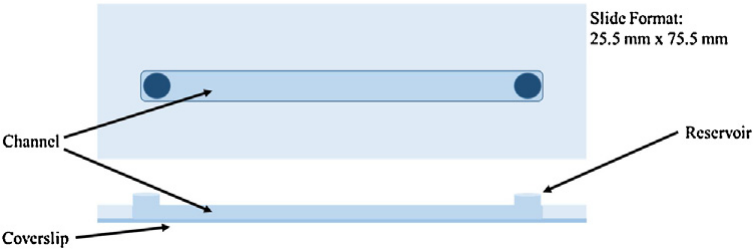
A schematic of the Ibidi Sticky Slide I^0.4^ Luer Lock slide with #1.5 IbiTreat polymer coverslip (#80178 and #10814, respectively, Ibidi, Martinsried, Germany) used in this study. The slide was made from a derivative of polyethylene (personal communication with Ibidi). The coverslip was treated with IbiTreat from Ibidi, which aided cellular growth and adherence. The slide features two reservoirs into which cells and fluid shear stress were administered into the channel. The 50-mm-long channel had a width of 5 mm and a depth of 400 μm, which allowed cells to adhere to the base of the coverslip and let fluid flow across the top of them. Since the channel width-to-height ratio is 12.5 and the length-to-height ratio is 125, it is reasonable to approximate the channel as two horizontal, infinite parallel plates. Thus, for this geometry, the flow in the channel is only parallel to the top and bottom of the channel and along the length of the channel. Thus, a homogenous fluid shear stress was applied to the culture area.

Cells were seeded into the channel of the slide at 5 × 10^4^ cells/cm^2^ (Fig. 1). The slide was placed in the incubator for two hours under static conditions (no flow) to allow the cells to adhere to the base of the channel. Adherence was confirmed at the two-hour time point with phase-contrast microscopy. Aseptically, the complete growth media was aspirated from the channel and a solution of 1:1000 Hoechst 33258 (#116M4139V, Sigma) was pipetted into the channel. The slide was incubated for 3 min in the CO_2_ incubator, then the Hoechst solution was aspirated and replaced with complete growth media. A total of 12-ml complete growth media was aseptically added to each reservoir set and connected to the fluidic units inside the CO_2_ incubator. The “remove air bubbles” sample experiment was run to clear the system of air that could cause an irregularity in the shear rate over the cells.

### Cell perfusion

The Ibidi Fluidic unit (#10903) was connected to the Ibidi Pump (#10905) and controlled with Ibidi PumpControl software (v1.5.2). System calibration was done using a red perfusion set and Sticky Slide I^0.4^ specifications. The viscosity for the complete growth media was 1.03 cP (measured at 37 °C using Brookfield DVII + Pro, ULA Spindle set, data not shown). The calibration factor was also set using complete growth media at 37 °C following the Ibidi protocol. The experimental shear stress was 5-dyn/cm^2^ (shear rate 485 s^−1^, pressure 8.1 mbar, flow rate 3.68 ml/min). For laminar flow the switching time was set to 10 s. After the two-hour cell adherence period, the slide was connected to the fluidic unit. Laminar, unidirectional flow was started at 5-dyn/cm^2^ for 24-h.

During the 24-h shear period, the following three solutions were prepared: (1) 1% (w/v) Paraformaldehyde (PFA) (#158127, Sigma-Aldrich) in PBS (#14190144, Gibco); (2) 2.5 M Glycine (BP381, Fisher Bioreagents) in Milli-Q type I ultra pure water; and, (3) 0.25% Trypsin + EDTA (#15400054, Thermo Fisher Scientific) in PBS (#14190144, Gibco). At the conclusion of 24-h shear period, the slide was removed and imaged using phase-contrast microscopy to visualize changes in cell and nuclear morphology.

### Cell fixation under shear

After the 24-h shear period the cells were fixed *in situ*. The perfusion set attached to the fluidic unit was replaced with new fixation perfusion set filled with 12-ml of the 1% (v/v) PFA solution. The “remove air bubbles” sample experiment was run to clear the fixation perfusion set of air that could cause an irregularity in the shear rate over the cells. The slide was carefully re-attached to minimize the introduction of air bubbles and the experimental shear stress (5 dyn/cm^2^) was resumed for 10 min.

### Quenching the fixation

After fixation, the slide was removed from the CO_2_ incubator and placed in the biological safety cabinet (BSC). Fixation was quenched by pipetting the 2.5M Glycine solution directly into the flow chamber. Glycine was pipetted into one reservoir, following the direction of flow during perfusion, and excess solution was aspirated from the other reservoir (following Ibidi Application Note 03: 2a Continuous Medium Exchange). This process was repeated five times to ensure the complete removal of PFA.

The slide was imaged using fluorescence microscopy to ensure the cellular morphology was maintained during fixation. The fixation perfusion set in the CO_2_ incubator was replaced with new quenching perfusion set filled with 12-ml 2.5M Glycine and the “remove air bubbles” sample experiment was run. Following imaging, the slide was reconnected to the fluidic unit and the experimental fluid shear stress (5 dyn/cm^2^) was resumed for 15 min.

### Cell dissociation and collection

After quenching, the slide was removed from the fluidic unit and the quenching perfusion set was replaced. A new dissociation perfusion set was filled with 12-ml 0.25% Trypsin + EDTA. The “remove air bubbles” sample experiment was run to remove air in the system. The slide was placed in the CO_2_ incubator and connected to the fluidic units. The experimental flow rate was resumed for 5 min to dissociate the cells from the base of the channel.

Fluorescence microscopy was used to confirm cell dissociation from the channel and to analyze the morphology of the nuclei after exposure to Trypsin to dissociate the cells from the coverslip. The cell solution was then collected by running laminar flow through the fluidic units for a long-time scale (switching time 60 s). The system ran through two cycles and cleared the perfusion set of the cell suspension and collected it in one reservoir. The fluidic unit was then stopped, and the perfusion set clamped off. The reservoirs were emptied into a 50-ml centrifuge tube and spun down (275 × *g*, 10 min, room temperature) to pellet the cells. The supernatant was discarded, and the pellet was suspended in PBS and stored at −20 ^○^C.

### Microscopy and image analysis

**Fig. 2:**
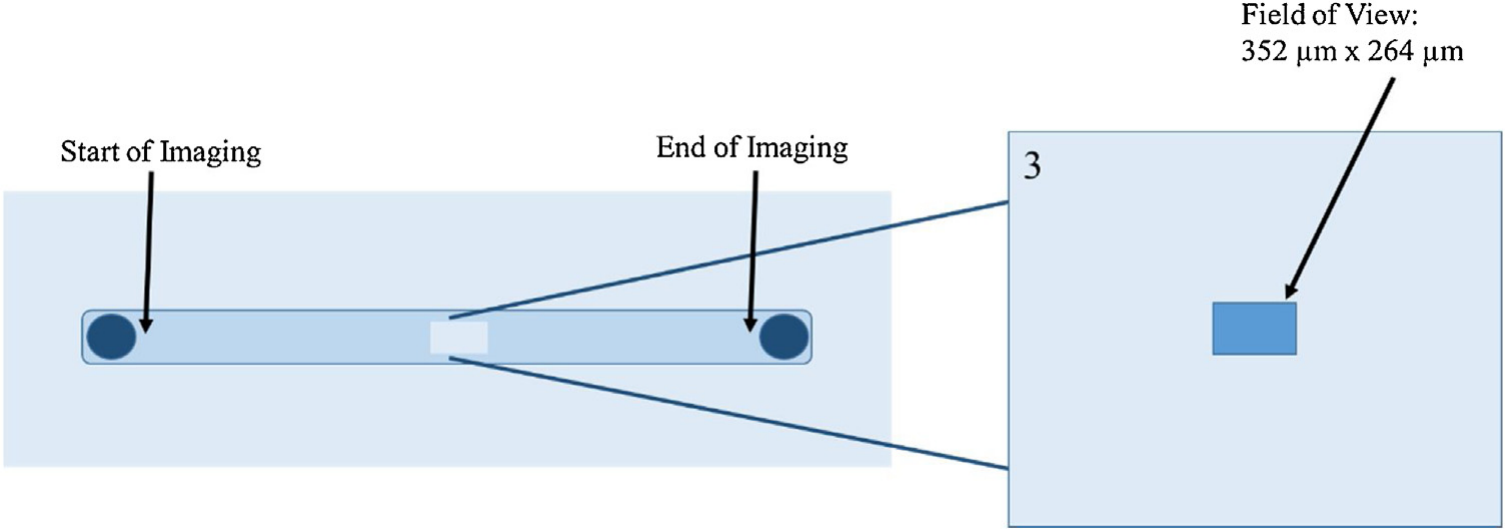
The image acquisition protocol used. Ibidi recommended that all observations should be made at a distance away from the side walls comparable to the height of the channel to ensure that observations are made where the fluid shear stress is homogenous (Ibidi Application Note 11). The height of the channel used in this study is 0.4 mm. Therefore, five equally-spaced and equal-sized areas (field of views) were marked along the center of the channel (∼2.5 mm from the side walls or ∼6.25 channel heights from the side walls). A single fluorescence image was acquired at each field of view. The camera’s field of view was 352 μm × 264 μm. The imaging process was started at the left edge of the channel (∼5 mm from channel inlet), where flow was initiated on the cells, and ended at the far-right edge at the end of the region of flow (∼5 mm from channel exit).

A Leica DMI 6000B inverted microscope and HCX PL FLUOTAR 20x/0.40 Corr PH1 objective were used to acquire phase-contrast and fluorescence images of the cells. Fluorescence image acquisition also used a Mercury-arc lamp (#EL6000, Leica) and DAPI ET filter cube (#11504203, Leica). The channel was divided into 5 equally-spaced sections along the flow direction and marked as the imaging locations (Fig. 2). Images were acquired with a Leica DFC345 FX camera with a field view of 352-μm × 264-μm. Phase-Contrast images were acquired both before and after perfusion, and fluorescence images were acquired after quenching and after dissociation. Images were taken at hours 0, 8, 16, and 24, as well as after fixing, after quenching, and after dissociating the cells. Since there were fewer cells in each FOV after dissociation, a total of 15 images were collected along the center of the channel. These additional images allowed for a greater number of nuclei to analyze.

**Fig. 3:**
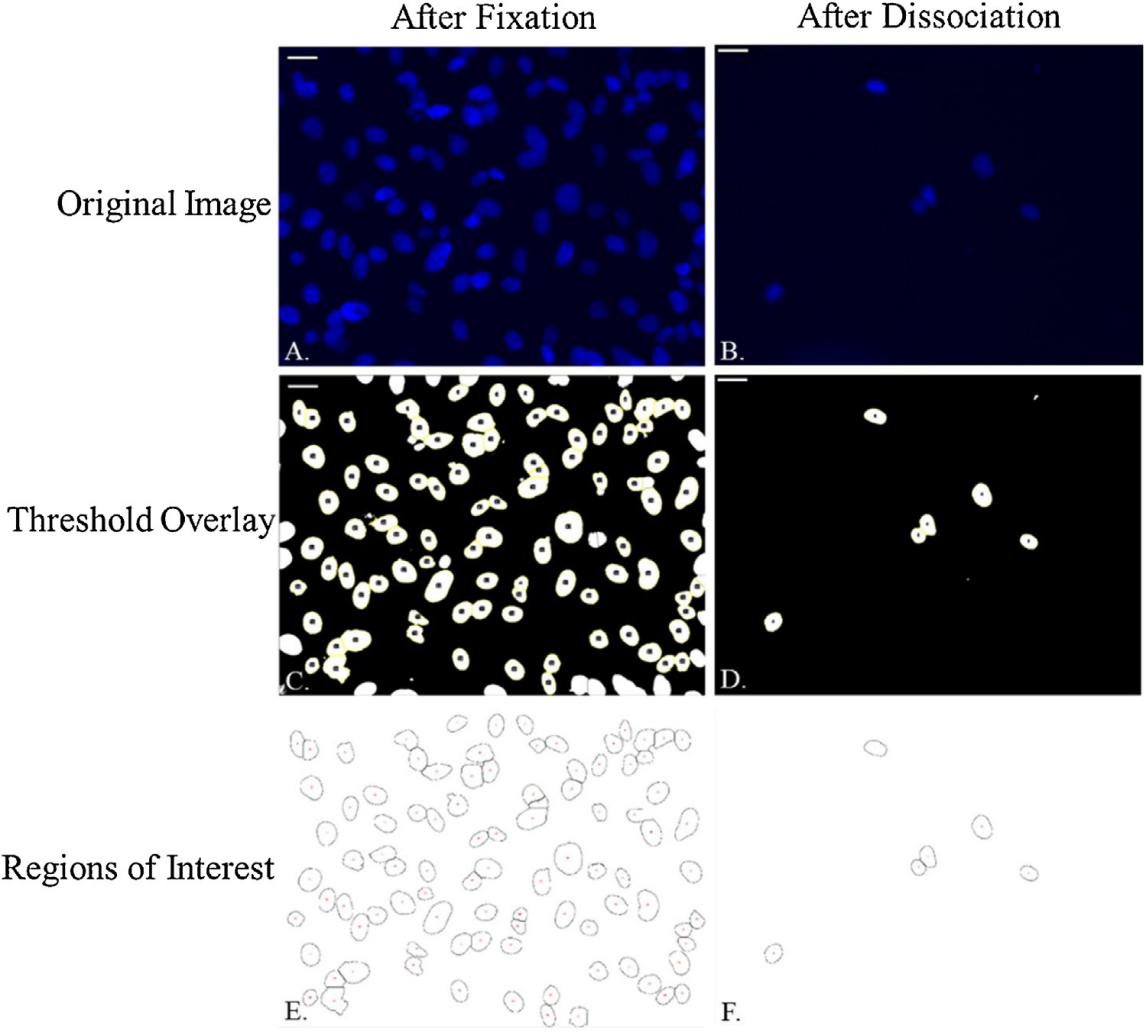
Representative images illustrating the image processing workflow. Image processing was performed the same way for all images (one image from each treatment is shown here). The original images (A and B) were converted to 16-bit gray-scale images, thresholded automatically, and then watershed segmented to isolate individual nuclei. After processing the images, an overlay was created that outlined the nuclei that would be used for data analysis (C and D). The regions of interest were isolated and analyzed, measuring the aspect ratio, area, perimeter, and circularity of each nucleus (E and F). From these data, there was no statistically significant difference in the aspect ratio of the nuclei before dissociation or after dissociation. Scale bar = 25 μm.

Images were analyzed using Fiji (ImageJ 1.52 g; Java 1.8.0_66) for image processing and MATLAB R2017a for statistical analysis (Fig. 3). The images collected after fixation (*n* = 5) and after dissociation (*n* = 15) were compared to determine if a statistically significant difference in nuclear shape was present after dissociation. Images were segmented following a standard threshold and watershed separation process. First, the scale was set to account for the field view the images were taken in and to change the unit to microns (pixel aspect ratio: 1.0; unit of length: micron; scale: 4.5455 pixels/μm). Then, images were opened in Fiji and converted to 16-bit images. Next, a Gaussian Blur filter with a radius of 1 μm was applied. Auto Thresholding (v1.17) under the default setting was run with white objects on a black background to create a binary image of isolated nuclei. To separate nuclei, the binary watershed segmentation was run. Finally, an erode function was used to distinguish nuclei that were in close proximity. The nuclei were analyzed and through the Regions of Interest (ROI) menu. The following exclusion criteria was used to exclude nuclei from the ROI and data analysis: (1) nuclei that were not completely in the FOV; (2) nuclei that did not watershed; or, (3) nuclei that were smaller than 100 μm^2^ or larger than 100,000 μm^2^. Morphological data on each nucleus, denoted as an ROI by Fiji, was exported for statistical analysis. Using MATLAB 2017a, a 2-sample *t*-test (α = 0.05) was run to compare the area, circularity, perimeter, and aspect ratio of each nucleus from the fixation images (*n* = 404) to those same parameters of each nucleus from the dissociation images (*n* = 22).

## Method validation

### Ibidi workflow

In this study, the development of a robust protocol to fix and remove cells after exposure to fluid shear stress was characterized. The initial set-up of the Ibidi Fluidic unit within the CO_2_ incubator as well as cell culturing protocol followed the outlined steps from Ibidi (Ibidi Pump System Instructions v. 1.5.2). Cells were fixed *in situ* to maintain any morphological change that may have occurred due to cellular exposure to fluid shear stress during culture. Based on qualitative data collected over the duration of the experiment, there was no cell viability decrease during the process. The cells were seeded at 5 × 10^4^ cells/cm^2^ and after 24-h had reached 100% confluence (approx. 200,000 cells/slide).

### Nuclear morphology

Nuclear morphology was analyzed using Fiji. The nucleus was segmented out of the image for analysis following the previously mentioned protocol. The ROI data for each nucleus was exported for statistical analysis. Individual ROI data were reviewed in comparison to the original images and was excluded from the data set if it met the following exclusion criteria: (1) the nucleus had non-distinct edges as classified by a noticeable blurring around the cell that would indicate the nucleus was not in focus; (2) failure of the processing protocol to appropriately watershed at the junction of two or more nuclei; and, (3) an over-saturation of the DAPI fluorescence signal that could affect the detection of the nuclear shape. Following this exclusion criteria, a total of *n* = 404 fixation nuclei and *n* = 22 dissociation nuclei were analyzed. The mean aspect ratio for the nuclei after fixation and after dissociation was, respectively, 1.43 and 1.50. Additionally, the nuclear area was 187.10 μm^2^ (after fixation) and 192.24 μm^2^ (after dissociation) with a perimeter of 52.58 μm (after fixation) and 53.52 μm (after dissociation) (see Table 1).

**Table 1:** The mean value and standard deviation of nuclear area, perimeter, circularity, and aspect ratio after fixation (*n* = 404) and after dissociation (*n* = 22). Since the cells were dissociated from the coverslip, the 22 cells imaged after dissociation may not be a part of the 404 cells imaged after fixation; however, all cells were exposed to the same conditions. No statistically significant difference was found between the area, perimeter, circularity, and aspect ratio of the cells after fixation and after dissociation. Therefore, the nuclear morphology is not affected by this method of cell dissociation after exposure to flow. Note – The tabulated values were rounded at the second decimal place.

Nuclear Morphology After Fixation and After Dissociation					
	Number	Area (μm^2^)	Perimeter (μm)	Circularity	Aspect Ratio
Fixation	404	187.10 ± 76.90	52.58 ± 10.04	0.82 ± 0.05	1.43 ± 0.22
Dissociation	22	192.24 ± 55.30	53.52 ± 7.02	0.83 ± 0.08	1.50 ± 0.52

In MATLAB, a 2-sample *t*-test for equal means, without assuming equal variances was done to determine if there was a significant difference in nuclear shape before and after the cells were dissociated. Data included the mean of the aspect ratio for *n* = 404 nuclei after fixation and *n* = 22 nuclei after dissociation. There was no expectation that the same nuclei were imaged before and after dissociation, however, all cells received the same shear treatment and were expected to show uniform results. The null hypothesis was that there was no difference in aspect ratio before or after dissociation. At α = 0.01, *p* =  0.9759 which failed to reject the null hypothesis. Additionally, using the 2-sample *t*-test for the area, circularity, and perimeter did not yield a significant difference in results between the cells after fixation and after dissociation. Based on these results, there was not a statistically significant difference between the nuclear morphology before and after cell dissociation in regard to area, perimeter, aspect ratio, or circularity.

## Method summary

The purpose of this experiment was to present a method for fixing adherent sheared endothelial cells *in situ* and collecting them under the same shear forces as during the experiment for Hi-C analysis. This method could allow for cells to be collected without causing a change in the nuclear and cellular morphology developed during the exposure to fluid shear stress. Current methods for removing adherent cells fixed *in situ* require the use of a cell scraper, which could introduce changes to the cellular morphology and is not a viable option to collect cells grown in micro- to nano-scale channels.

In the development of this protocol, standard cell culture methods were taken into account to ensure the viability of the cell culture and produce repeatable results. The perfusion shear stress of 5 dyn/cm^2^ was chosen to mimic the blood flow within human microcirculation [3]. The cells were kept at 37 °C to maintain ideal growing conditions. A preliminary study was done to determine the type of dissociation reagent that would be necessary to remove the adherent cells, as flow without a dissociation reagent was not able to dissociate the cells from the growth surface. The reagents considered in the preliminary study included 0.5% Trypsin, 0.5% Trypsin + EDTA, 0.25% Trypsin + EDTA, and 1X TrypLE. Based on standard cell viability assays (*i.e.*, Trypan Blue, live-dead cell count using a hemocytometer) and the number of cells removed from the culture dish, the 0.25% Trypsin + EDTA was selected for this study. This treatment balanced cell viability and cell dissociation more effectively than the other reagents, and it is traditionally used in studies involving the culture of endothelial cells.

For the method proposed here, cells were seeded into the Ibidi Sticky Slide I^0.4^ Luer Lock slide with #1.5 IbiTreat polymer coverslip at a seeding density of 5 × 10^4^ cell/cm^2^ (approx. 85% confluent) and reached 100% confluent with ˜200,000 cells/slide after 24 h. The perfusion process with complete growth media followed by 1% (w/v) PFA, 2.5 M Glycine, and Trypsin + EDTA did not cause a visible decrease in cell viability or overall number of collected cells. During each of these treatments, the same fluid shear conditions were used in order to maintain the changes in nuclear morphology and chromatin structure that were developed during the culture process. After dissociation, the cells were collected and centrifuged at 275 × *g* for 10 min at room temperature before being suspended in PBS. The slides were viewed under phase contrast microscopy to determine if the cells dissociated from the coverslip. A sample of the PBS solution included cells visible under phase contrast microscopy (10×) and a review of the slide the cells were cultured on revealed a detachment of ˜95% (qualitatively) of cells from the coverslip. Cells remaining in the channel were only visible on the peripheral edges where the coverslip adhered to the slide. Under a low-power objective (10×), these edges are not smooth and can cover cells so that they may not be affected by the fluid flow. Based on these results, we concluded that the cell harvesting protocol was effective at dissociating and collecting the cells post *in situ* fixation.

The images collected during the study were processed in FIJI to segment individual nuclei and determine the nuclear area, perimeter, circularity, and aspect ratio. When analyzed by the 2-sample *t*-test, the null hypothesis that there was no difference in cell shape as related to aspect ratio could not be rejected. This led to the conclusion that the *in situ* fixation and shear removal strategy were effective at maintaining the nuclear morphology. Additionally, there was not a statistically significant difference in the area, perimeter, and circularity of the nuclei between fixation and dissociation. Thus, this method provides a promising solution for the removal of adherent cells fixed *in situ* that could be used in applications beyond the test case.

When imaging the dissociated nuclei, the *z*-plane was kept fixed, presenting a challenge in collecting images that were in focus as the cells floated freely in the dissociation reagent. Imaging at the low objective power (20×) did not allow for the opportunity to image individual nuclei in every field of view. Because of this, the images contained in focus, out-of-focus, and over-saturated nuclei. After processing the images in the same manner as the images after fixation, it was necessary to develop an objective exclusion criteria. Following the exclusion criteria explained previously, the total number of dissociated nuclei imaged reduced from 35 to 22. The removal of out-of-focus and over-saturated nuclei that could not be confidently segmented eliminated outliers. This exclusion process was done objectively and was independently reviewed by multiple people to eliminate potential bias.

Future experiments using this protocol will not require the use of Hoechst to stain the nuclei, nor imaging the nuclei. As a result, the protocol can be done with limited interruption to fluid flow over the cells beyond the time required to exchange the reservoir and perfusion sets. This protocol can be used with any type of flow chamber or channel-based cell culture system, any pump system, any type of flow (*e.g.*, steady, pulsatile, or oscillatory), any physiologically-relevant shear stress, and is not limited to the Ibidi pump system used in this experiment. To demonstrate the protocol, we used a fluid shear stress of 5 dyn/cm^2^ — a fluid shear stress near the lower of end of physiologically-relevant shear stress and one that mimics blood flow in the microcirculation. We are confident that the cell harvest protocol outlined will work for any physiologically relevant fluid shear stress values including higher shear stress higher than 5 dyn/cm^2^. In fact, we believe the cell harvesting will be as effective (measured by the percentage of cells successfully removed from the channel) —if not more— for higher fluid shear stresses. The versatile nature of this protocol will allow it to be used in a variety of capacities, not limited to the Ibidi and vascular flow experiments. We believe this protocol will be a benefit for the collection of cells from any enclosed flow chamber for Hi-C analysis that will maintain intact chromatin structure.

Overall, this method effectively addresses the concern of removing adherent cells fixed *in situ* within an enclosed flow chamber without the use of a cell scraper. Throughout the duration of the process, the cells were exposed to uniform fluid flow. The pump operated with the same conditions during the shearing, fixing, quenching, and dissociating processes, setting it apart from methods that utilize a cell scraper. By dissociating the cells at the same fluid shear rate as they were cultured and fixed, we believe this method mitigates the risk for potential damage to the chromatin structure. Other cases that could benefit from the use of this method may include studies examining the effect of shear on other types of endothelial cells or in preparation for Hi-C and similar analysis on cells in microfluidic devices or parallel plate flow chambers. The universal application of this method fills a gap in the ability to dissociate and analyze adherent cells fixed *in situ*.
